# Comparison of Naturally Conceived and IVF-DZ Twins in the Netherlands Twin Registry: A Developmental Study

**DOI:** 10.1155/2011/517614

**Published:** 2011-11-03

**Authors:** Catharina E. M. van Beijsterveldt, Meike Bartels, Dorret I. Boomsma

**Affiliations:** Department of Biological Psychology, VU University, 1081 BT Amsterdam, The Netherlands

## Abstract

In a large set of twin pairs, we compared twins born after IVF to naturally conceived twins with respect to birth characteristics, growth, attainment of motor milestones, and emotional and behavioral problems. Twin families were registered with the Netherlands Twin Register. We included 1534 dizygotic (DZ) twins born after IVF, 5315 naturally conceived (NC) DZ twins, and 1504 control NC DZ twins who were matched to the IVF twins based on maternal age, maternal educational level, smoking during pregnancy, gestational age, and offspring sex. Data were obtained by longitudinal surveys sent to fathers, mothers, and teachers at ages 1, 2, 3, 7, 10, and 12 years. Results showed no differences in growth, in attainment of motor milestones, and in behavioral development between IVF and matched NC twins. It can be concluded that for nearly all aspects, development in IVF and NC children is similar.

## 1. Introduction

In the Netherlands, the number of children born after assisted reproductive technologies (ART) including in vitro fertilization (IVF) and intracytoplasmic sperm injection (ICSI) is rapidly increasing. In 1996, 1 in 77 newborns was born after IVF or ICSI treatment and in 2005, this had increased to 1 in 43 [1, 2]. The first IVF treatment in the Netherlands was in 1983, and ICSI was introduced in 1994 [3]. Nowadays, ICSI is often used simultaneously with IVF treatment [2]. The introduction of ART was accompanied by an increase in the number of multiple births. Between 1987 and 1994, the percentage of multiple births after IVF/ICSI treatments fluctuated around 25% [4]. This percentage dropped to 18.5% in 2005 but a significant proportion of ART treatments still results in a twin pregnancy. There is no doubt that twin pregnancies have a higher risk of complications compared to singleton pregnancies. However, only a few studies have compared the long-term development between twins following IVF/ICSI treatment and twins following natural conception (NC) [5]. The main aim of the present study was to investigate the short- as well as the long-term development of twins born after IVF/ICSI treatment and after NC.

Previous research on perinatal and obstetric outcomes of twin pregnancies after assisted reproduction has produced mixed results. The comparison of the outcomes of IVF/ICSI twin pregnancies and NC twin pregnancies is complex because IVF/ICSI mothers are older than mothers of NC twins, are more often primiparous, and have a history of infertility problems, all factors that may negatively influence perinatal and obstetric outcomes [6]. In addition, studies that included both MZ and DZ twins as controls may be biased as there are more adverse effects in MZ pregnancies [7]. Even when comparisons are restricted to DZ twins only, studies have shown large differences in outcomes. Some studies reported a higher rate of preterm birth and lower birth weight in IVF/ICSI twins [8–10], whereas others reported no differences in perinatal outcomes between the two groups [11–13]. The question thus remains whether the adverse perinatal outcomes are due to maternal characteristics or due to the IVF procedure itself. A recent meta-analysis of perinatal risks in twins [14], which selected studies that matched or controlled for maternal age and often other factors, showed that IVF twins had an increased risk of preterm birth and low birth weight compared to NC twins.

In recent years, a growing number of studies investigated the longer-term development in growth, health, and psychosocial development of IVF/ICSI children [5, 15, 16]. The developmental trajectories in IVF/ICSI children could be different because of the IVF procedure itself, as consequence of the infertility problems or as an effect of problems in the perinatal period, such as lower birth weight and shorter gestational age. There is also evidence that parents of IVF children and NC children differ with respect to parental attitudes, parental concerns, and educational styles [17–20].

Most research on IVF-related outcomes is done in singletons, and data on development of IVF twins are limited [5, 21]. In the first 3 years of life, lower weight and height for IVF singletons compared to controls have been reported [22, 23], with the most pronounced differences during the first 6 months. However, studies comparing IVF and NC twins found no growth differences in the first years of life [23, 24]. For growth between ages 5 and 18, no differences in weight and height were seen between IVF singletons and NC controls born to subfertile parents [25, 26], although Ceelen et al. [25] found evidence that IVF children had more peripheral body mass and fat as compared to controls [25]. For motor development, there were no differences between IVF/ICSI children and NC children during childhood [23, 27–30]. Studies comparing behavioral and emotional problems between IVF and NC children showed mixed results. Up to age 9, IVF singletons showed normal behavior and socioemotional functioning [17, 31–33]. Parents of IVF adolescents even reported fewer externalizing problems [31, 34]. Parents and teachers of IVF singletons reported more withdrawn/depressed behavior than the parents of NC singletons [34], but when these children reported on their own behaviors [35], no differences were observed in behavioral functioning between the IVF and the control group [35]. In twins, parental ratings of externalizing and internalizing problem behaviors of 5-year olds were similar in IVF and NC twins. Teacher ratings of the twins' behavior did not differ between IVF and NC twins [36]. Taken together, the current data suggest that IVF singletons and twins show normal psychosocial development during childhood. 

Up until now the short- and long-term development of IVF/ICSI children has mainly been studied in singletons. Because a significant proportion of IVF pregnancies results in a twin pregnancy, it is important to examine whether there are differences in development between twins after fertility treatment and NC twins. Comparing IVF twins to control samples of singletons may introduce bias as twins are at higher risk than singletons for low birth weight, low gestational age, and developmental delays. In this study we compare the development of IVF twins to carefully matched control twins. We look at perinatal outcomes, growth, motor development, and behavior problems during childhood. Because the proportion of MZ twins is low following IVF/ICSI conception, only DZ twins were included. The IVF and the NC DZ twins were matched on birth cohort, maternal age and educational level, smoking behavior during pregnancy, and gestational age of the twins.

## 2. Methods

### 2.1. Participants

The data on mode of conception and development measures in twins come from a longitudinal study designed to examine the genetic and environmental influences on the development of behavioral and emotional problems in twins from birth onwards. The twin families are volunteer members of the Netherlands Twin Register (NTR) maintained by the Department of Biological Psychology at VU University in Amsterdam [37–39]. The NTR recruits families with twins a few months after birth. Depending on birth cohort, between 25 and 40% of all multiple births in The Netherlands are registered by the NTR. For the present study, data obtained at ages 1, 2, 3, 7, 10, and 12 years were included for twins born between 1990 and 2000.

Data on mode of conception, mode of delivery, age at birth, gestational age, birth weight, birth order, sex of twins, and smoking behavior of both parents during pregnancy come from survey-1 which is collected after parents register their twins (age < 1 years). Information on maternal educational level was obtained at age 3 of the twins (survey-3), and if missing, educational level was supplemented with information obtained at ages 7 (survey-7) or 10 (survey-10). 

For 11708 twin pairs complete data on the variables used for matching were available (i.e., mode of conception, gestational age, age of mother at birth, smoking behavior during pregnancy, zygosity, and maternal educational level). There were 9001 twin pairs who were born following natural conception, 1606 pairs born following IVF/ICSI (at least 288 pairs after ICSI), and 1101 pairs born after ovulation induction. For the analyses we excluded twin pairs conceived by ovulation induction and all MZ pairs (*N* = 72 for the IVF/ICSI group; *N* = 3686 for the NC group). Information on zygosity of 808 same-sex twin pairs was based on blood group/DNA group polymorphisms. For the remaining same-sex twin pairs (*N* = 6998), zygosity was assessed using items about physical similarity and frequency of confusion of the twins by family and strangers [40], collected in surveys at 3, 5, 7, 10, and 12 years.

In the analyses, there were 1534 DZ IVF/ICSI twin pairs (1606 minus 72 MZ twin pairs); these are referred to as IVF twins throughout the paper. From a total of 5315 NC DZ twin pairs, a control group of NC DZ twin pairs was formed by matching for birth cohort, gestational age, age of mother at birth, smoking behavior during pregnancy, zygosity, and maternal educational level (*N* = 1504).

### 2.2. Measures

#### 2.2.1. Mode of Conception

Survey-1 included one question about the use of hormonal preparations. Possible answers were (1) no hormonal preparations, (2) oral contraceptives before getting pregnant, (3) ovulation induction, and (4) ovulation induction in combination with IVF. Endorsement of more than one answer was possible. In 2005, a 2-page survey with questions about familial twinning, fertility, and twin pregnancy was sent to all mothers of twins who were registered with the NTR [41]. This survey included one item on mode of conception, with the following answers: (1) naturally conceived, (2) IVF, (3) ICSI, (4) IUI, (5) ovulation induction, or (6) other, with additional space for comments [42]. For mothers who only returned survey-1, we formed the groups of naturally conceived twins, IVF twins, and twins born after ovulation induction. For about 70% of the twin pairs, the mother returned both surveys. For these twin pairs we could make an additional distinction between IVF and ICSI twin pairs.

#### 2.2.2. Motor Milestones

In survey-2, mailed out when the twins were 2 years old, the mother was asked to report the age at which certain motor milestones were reached (turning over from back to belly (turning), sitting without support (sitting), crawling on hands and knees (crawling), standing without support (standing) and walking without support (walking)) [43, 44]. With survey-1, mothers received a memory aid to track the motor milestones. For 476 children (from 238 twin pairs), the mailed survey data were compared with monthly telephone interview data collected from the mothers on the time which motor milestones were achieved. With exception of “standing”, no differences in times were found between the two assessment methods [43].

#### 2.2.3. Behavior Problems Rated by the Parents and Teachers

At age 3, externalizing and internalizing behavior problems were assessed using the CBCL/2-3 [45]. Both parents were asked to rate the behavior of the children for the preceding 6 months on a 3-point scale. The CBCL includes two broad categories of problem behaviors: externalizing behaviors (including the syndromes: aggressive behavior, oppositional and overactive problems) and internalizing behaviors (including the syndromes: anxious and withdrawn/depressed). The syndromes are constructed for the Dutch population [46] and comparable with the syndrome scales as developed by Achenbach [45].

Behavior problems were measured at ages of 7, 10, and 12 years using the CBCL/4-18 [47]. The scales overlap with the CBCL/2-3 to a large extent. Externalizing behaviors include the syndrome scales: rule breaking and aggressive behavior and internalizing behavior includes withdrawn, somatic complaints, and anxious/depressed behavior. In addition, data from the Attention Problems scale were analyzed.

After consent was obtained from parents, teachers of twins were asked to fill in a questionnaire about the twins' behavioral problems. Teachers were required to have known the children for at least 3 months. At the ages of 7, 10, and 12 years, teachers rated behavioral problems using the Teacher's Report Form (TRF [48]). The TRF scales are comparable with the scales of the CBCL4-18, although item content can differ slightly.

#### 2.2.4. Educational Level

Maternal education level was measured on a 13-point scale, ranging from primary education to postdoctoral education. Educational level was classified into three categories (low, middle, and high).

#### 2.2.5. Growth

Mothers of twin pairs were asked to report offspring height and weight in the surveys at ages 1, 2, 3, 7, 10, and 12 years. Data were converted to Standard Deviations Scores (SDS) by comparison of weight and height to the general population using the software package growth analyzer 3.5 containing the Dutch reference growth charts for the general population from 1997 [49, 50]. The SDS scores indicate by how many standard deviations the relevant measurement differs from the mean of the Dutch reference growth charts.

### 2.3. Statistical Analyses

The data were analyzed using SPSS version 17.0 (statistical packages for social sciences). As first step we compared the maternal and demographic characteristics of twins in the IVF group versus all DZ NC twins. In a second step, the IVF group was compared to a group of matched NC twins. Matching of IVF and NC pairs was done by using the “duplicate case” option in SPSS. Differences in proportions for parental and birth characteristics between IVF and NC twins were tested using chi-squared tests. For the continuous dependent variables, ANOVA was used to compare the means for maternal and twin pair characteristics. The mixed models procedure and generalized estimating equations (GEE; SPSS [51]) were used for the comparisons of the characteristics of the individual children between IVF and matched NC groups. In twin data observations are not statistically independent as there are two children from the same family. Using mixed models, and GEE it is possible to adjust for this dependency in the data of twin pairs. To evaluate the importance of significant findings, the effect size (Cohen's *d*) was computed. This was done by computing the difference between estimated means divided by the square root of the standard deviations of the 2 groups. An effect size of 0.20 is considered small, of 0.50 moderate, and 0.80 large. To correct for multiple testing and to determine the significance of the results, Bonferroni correction was applied by dividing the significance level by the number of independent traits in each developmental domain.

## 3. Results

### 3.1. Parental, Birth, Child Characteristics

First, parental characteristics were compared between the IVF twins and the unmatched NC twins. Results are given in Table 1. In the IVF group, both mothers and fathers were older at the birth of the twin pair compared to the parents of unmatched NC twins. Mothers of unmatched NC twins smoked more often during pregnancy than the mothers of IVF twins. No differences were found in educational level between the two groups of mothers. This reflects the fact that in The Netherlands IVF treatment is paid for by health insurance, which is obligatory with private health insurance companies, and IVF is equally accessible to parents from different socioeconomic backgrounds. Gestational age was shorter and more preterm births were observed in the IVF group compared to the unmatched NC group. In addition, mothers of unmatched NC twins were taller and weighed more than mothers of IVF twins. No differences were found in weight gain during pregnancy between the two groups.

To investigate the possible risks of IVF on health, growth, motor development, and problem behaviors we obtained a control group of 1504 twin pairs who were matched on maternal age, gestational age, educational level, zygosity, and smoking behavior. Comparisons of the IVF pairs with the NC pairs matched on maternal and birth characteristics are given in Table 1. After matching, there is still a large difference between the IVF and matched NC group in the number of older sibs. In contrast to the matched NC group, for the vast majority of IVF mothers the twin birth was the first birth.

### 3.2. Birth Weight, Mode of Delivery, and Hospital Admission

Both first- and second-born IVF twins had lower birth weights and lengths than matched NC twins, but the effect was augmented in second-born twins. However, the proportion of children with a birth weight lower than 1500 grams is not different between second-born IVF and matched NC controls. In addition, birth weight discordance was found to be larger in the IVF twins than in matched NC twins. When the analyses were restricted to primiparous mothers, the differences in birth length and weight and birth weight discordance disappeared, with the exception of birth weight of the first born twin (*P* = 0.023). As shown in Table 2, IVF twins were more often delivered by caesarean section compared to control twins. The difference in frequency of caesarean sections between the 2 groups remained when limiting the analyses to primiparous mothers. In the IVF group the rate was increased to 40.6% and in the control group to 33.8%. 

Admission rate to an incubator was the same for IVF and matched NC twins, but the number of days in an incubator was higher in the IVF group compared to the matched NC group. In addition, the proportion of twins that remained in an incubator for longer than 1 month was larger in the IVF group compared to the matched NC group. After controlling for parity, the difference in time in incubator was no longer statistically significant. At age 3, the proportion of children with a hospital admission was similar in IVF and matched NC twins.

### 3.3. Motor Milestones and Growth

We found significant differences for the ages at which three motor milestones were attained, with IVF twins doing better than matched NC twins (see Table 3). The effect sizes were 0.11, 0.12, and 0.08 for sitting, standing, and walking, respectively. 

The SDS's for height and weight across ages 1 to 12 years are given in Table 4. At age 1, both IVF and matched NC twins were 0.5 SD smaller and weighted less than children of the same age in the Dutch population. Until age 7, twins remained smaller than children in the Dutch population. After the age 7, the twins were similar in height. For weight, the pictures differ to that for height. Until the age of 12, the twins remained smaller than their peers in the Dutch population. These trends were the same for the IVF group and the control group (at a significance level of 0.008 (0.05/6)). Thus, growth patterns did not seem to differ between IVF and matched NC twins.

### 3.4. Behavioral Problems

The results for behavioral problems are presented in Table 5. Univariate analyses did not reveal any differences between the IVF and the matched NC twins. At all ages and for all raters, the IVF and the matched NC twins had the same scores for internalizing and externalizing behavior, and attention problems. At ages 3 and 7, IVF children tended to have slightly higher internalizing scores as rated by their mother and by their teachers. However, the effect sizes were very small (0.06, 0.08, and 0.09, resp. for internalizing problems at age 3 as rated by mother, and at age 7 as rated by mother and teacher).

## 4. Discussion

To date, there are limited data on the development of IVF/ICSI twins. Therefore, the main purpose of this study was to compare the development of IVF twins with that of naturally conceived (NC) twins with respect to motor milestones, growth, and behavioral problems during childhood. To control for possible confounding factors, only DZ twins were included, and the NC control group was matched on birth cohort, maternal age and educational level, smoking behavior during pregnancy, and gestational age. Although the perinatal outcomes appear to be slightly worse in IVF twins, the course of postnatal development is very similar in IVF and NC twin children. 

For the IVF pregnancies, maternal age was higher and more mothers were primiparous compared to NC pregnancies. Both factors increased the risks for adverse effects on perinatal outcomes. In our data, this was reflected in the higher proportion of preterm births in the IVF group compared to the NC twins. To control for these differences in demographic and maternal characteristics, we matched on these variables. After matching, the results suggest a slight effect of IVF/ICSI treatment on birth weight and length and time in incubator. This effect is probably due to parity. When only primiparous mothers were included in the analyses, effects were no longer significant. Our results agree with those of studies that controlled for chorionicity [11, 13] but not with a recent meta-analysis that suggested more preterm births and lower birth weight in IVF pregnancies [14]. Results among studies may differ because all the studies included different control variables. In the meta-analysis there was no adjustment for chorionicity, while this factor might be an important factor in the comparison of perinatal outcomes between IVF/ICSI and NC twins [13]. So, it seems that IVF pregnancies have a more adverse outcome, but it is an open question whether this is due to the IVF treatment itself. 

In agreement with other studies [7, 52], we found that caesarean sections (CSs) occur more frequently in the IVF group. Even after correction for parity, the rate of CSs is higher for IVF births than for NC births. This higher rate may reflect an increase in the deliberate choice for elective CSs in IVF pregnancies. As an IVF birth is often the first birth after a the history of infertility [53], both the physician and mother may be more worried about delivery than in NC pregnancies [52] and may more often plan a CS in advance. However, this does not entirely explain the increase of number of CSs in the IVF group. For a number of the deliveries in our study it was known whether the CS was elective or not. Among all CSs, we found that in the matched NC group of women of 35 years or older, 44% of the CSs were unexpected and 56% were agreed on beforehand. In the IVF group of older women, 57% of the CSs were unexpected, and 43% were elective CSs. The increased rate of CSs in the IVF group is partly due to deliberate choice for elective CS and partly to an increase of emergency CSs, at least in women aged 35 years or older. It cannot be excluded that IVF itself may be a risk factor for the increased rate of CSs. 

There are almost no studies that compared growth of IVF and NC twins. Until age 3, earlier studies found no evidence for differences in growth measures between IVF and NC twins [23, 24]. The present study investigated growth from birth to 12 years of age and found no evidence for differences in height and weight between IVF and matched NC twins. However, weight and height of the twins are lower than their peers in the Dutch population. Estourgie-van Burk et al. [54] described the growth pattern of twins from birth to 18 years of age. At the age of 4, twins were comparable on height with peers of the Dutch population. It seems that the twins in our study caught up at a later age. At age 7, the twins were still smaller than their peers. This may be explained by the relatively high rate of preterm births in both the IVF twins and matched NC group, as matching was done on gestational age. The same pattern seems to occur for weight. In the study of Estourgie-van Burk et al. twins had a SDS of −0.14 at age 4, while we observed an SDS of −0.30 at age 7. Weight at age 12 is still behind of that of children in the Dutch population, but height is similar to that of the children in the Dutch population. The different growth pattern for weight and height is also seen in the study of Estourgie-van Burk et al. [54]. The growth pattern of height and weight seems not to be different between IVF and NC control twins.

In agreement with other studies we did not observe a delay of the gross motor development in the IVF twins compared to the NC twins [23, 27–30]. A notable finding was that IVF twins achieved certain motor milestones earlier compared to NC control twins. The vast majority of the IVF twins are the first-born children after a period of involuntary childlessness. Earlier research showed that mothers of IVF twins were more emotionally involved with their child and interacted more with their child than mothers of NC children [20, 55]. Probably, parents of a first-born child push their child more to achieve motor milestones than parents of children with older siblings. To verify this in our study, we compared the attainment of motor milestones between children with and without older siblings. We found that children without an older sibling achieved the motor milestones sitting and walking significantly earlier than the children with an older sibling. 

We reported no differences in externalizing behavior and attention problems between the IVF and NC children. The results were found for both parental ratings and were further confirmed by the ratings of the teachers. The confirmation by the teacher ratings is important because the teacher is unaware of the means of conception. The results are in agreement with an earlier study with 5-year old twins [36] which showed no differences in externalizing, internalizing, and hyperactive behaviors between IVF and NC twins as rated by their parents and teacher. However, some studies with IVF singletons showed that parents reported less externalizing behaviors in IVF singletons as compared to NC singletons [31, 34]. A possible reason might be that mothers of IVF singletons were more emotionally involved and that these mothers were more prone to positively evaluate their child. At the younger ages, there was a trend for mothers and teachers to report more internalizing problems. More internalizing problems have been reported for IVF singletons at different ages during childhood [32, 34]. Again, this may result from overprotection by the mother, so that the child may show more anxious and withdrawn behaviors. It should be noted that in our study the effect sizes were very small and that the fathers did not report more internalizing problems for IVF children. It can be concluded that the behavioral development of IVF twins is similar to that of NC twins.

A limitation of the current study is that the results rely entirely on self-reports of the mode of conception. A study of Raj and Morley in 2007 [56] suggested that parents of twins showed less willingness to report about the mode of conception if there was no need for it. However, in a mailing sent to more than 20000 mothers of twins, we did not find evidence for less willingness to answer questions on mode of conception [42]. In the same study we found an agreement between maternal report and hospital records of 94%. A second limitation is that for a part of the IVF pregnancies it is not clear whether the pregnancy is achieved via IVF or ICSI. We have treated IVF and ICSI twins as one group, which may bias the results if the developmental outcomes differ between IVF and ICSI children. In a study that compared problem behaviors in 5 to 8 year-old singletons born after ICSI, IVF, and NC, no differences were found between these groups [57]. In the current version of our survey-1 we now make a distinction between IVF and ICSI, and in the future we may investigate whether there are developmental differences between IVF and ICSI children. 

In summary, children born after IVF develop similarly to NC children. Regarding growth, motor development, and behavior problems IVF twins do not differ from NC twins. Parents conceiving twins via IVF can assume that the development of their children will be similar to that of NC twins. Nonetheless, the risk of adverse obstetric outcome in IVF twin remains considerably higher than in NC twins and should not be underestimated [58].

## Figures and Tables

**Table 1:** Parental, birth, and child characteristics of IVF/ICSI DZ twin pairs, naturally conceived (NC) DZ twin pairs, and a group of DZ twin pairs matched for birth cohort, educational level, maternal age, gestational age, and smoking behavior during pregnancy (matched NC pairs).

	NC pairs *N* = 5315	IVF/ICSI pairs *N* = 1534	P*	Matched NC pairs *N* = 1504	P**
Age mother (y) (mean ± SD)	30.99 (3.78)	33.25 (3.44)	<0.001	33.08 (3.68)	0.185
Age father (y) (mean ± SD)	33.19 (2.44)	36.03 (4.68)	<0.001	35.01 (4.69)	0.001
Smoked during pregnancy (% yes)	1344 (25.3%)	264 (17.2%)	<0.001	283 (18.8%)	0.249
Educational level					
% Low	1999 (37.6%)	553 (36.0%)	0.515	544 (36.2%)	0.842
% Middle	2184 (41.1%)	651 (42.4%)		649 (43.2%)	
% High	1132 (21.2%)	330 (21.5%)		311 (20.7%)	
Gestational age (weeks) (mean ± SD)	36.84 (2.43)	36.40 (2.54)	<0.001	36.50 (2.43)	0.241
% >32 and <37 weeks	1539 (29.0%)	561 (36.6%)	<0.001	551 (36.6%)	0.847
% ≤32 weeks	326 (6.1%)	109 (7.1%)		99 (6.6%)	

Child sex (% boys)	5464 (51.4%)	1586 (51.7%)	0.775	1566 (52.1%)	0.775
Older sibs (%)	2199 (57.2%)	232 (21.5%)	<0.001	691 (63.3%)	<0.001
Height mother (cm) (mean ± SD)	170.39 (6.42)	169.91 (6.67)	0.012	170.49 (6.45)	0.017
Weight before pregnancy (kg) (mean ± SD)	69.13 (11.77)	68.52 (11.69)	0.077	69.40 (11.95)	0.042
Weight gain during pregnancy (kg) (mean ± SD)	16.41 (6.23)	16.35 (6.54)	0.782	16.24 (6.04)	0.630

Note: *P value based on tests comparing IVF and unmatched NC twin pairs **P value based on tests comparing IVF and matched NC twin pairs.

**Table 2:** Mode of delivery, birth weight and length, incubator, and hospital admission for IVF/ICSI twins and matched NC twins.

	IVF/ICS twins	Matched NC twins	P*
Delivery (% caesarean-section)	1085 (36.1%)	804 (27.2%)	<0.001
Birth weight (g) (mean ± SD)			
First born	2494 (557)	2560 (546)	0.001
Second born	2405 (563)	2507 (542)	<.001
Birth weight (% <1500 gr)			
First born	69 (4.5%)	44 (3.0%)	0.024
Second born	90 (5.9%)	66 (4.5%)	0.074
Birth weight discordance (mean ± SD)	13.01 (10.40)	12.05 (9.46)	0.009
% with ≥20% discordancy	338 (22.3%)	276 (18.8%)	0.016
Birth length (mean ± SD)			
First born	46.47 (3.73)	46.86 (3.58)	0.015
Second born	46.25 (3.83)	46.76 (3.51)	0.002
Incubator (% yes)	1566 (51.6%)	1454 (48.9%)	0.039
Incubator (days) (means ± SD)	11.77 (15.44)	10.16 (15.36)	0.044
Incubator: more than 1 month (% yes)	171 (11.8%)	109 (8%)	0.014
Hospital admission at age 3 (% yes)	422 (15.2%)	365 (13.2)	0.055

Note: *P value based on tests comparing IVF and matched NC groups.

**Table 3:** Mean and standard deviation (SD) of ages (months) at which motor milestones are attained for IVF/ICSI twins and matched NC twins.

	IVF/ICSI twins			Matched NC twins			P*
	*N*	Mean	(SD)	*N*	Mean	(SD)	
Turning	2535	6.3	(1.7)	2473	6.4	(1.8)	0.870
Sitting	2553	8.7	(1.8)	2501	8.9	(1.8)	0.008
Crawling	2296	9.7	(1.7)	2244	9.7	(1.7)	0.448
Standing alone	2533	12.2	(2.4)	2470	12.5	(2.6)	0.003
Walking alone	2632	14.8	(2.4)	2605	15.0	(2.4)	0.002

Note: *P value based on tests comparing IVF and matched NC groups.

**Table 4:** SDS height and weight for ages 1 to 12 years for IVF/ICSI twins and matched NC twins.

	IVF/ICSI twins			Matched NC twins			P*
	*N*	Mean	(SD)	*N*	Mean	(SD)	
Height							
Age 1	2571	−.42	(1.0)	2568	−.51	(1.0)	0.018
Age 2	2443	−.25	(1.1)	2421	−.32	(1.1)	0.064
Age 3	2262	−.13	(1.1)	2194	−.18	(1.0)	0.249
Age 7	1584	−.09	(1.1)	1543	−.03	(1.0)	0.237
Age 10	857	−.05	(1.0)	757	.04	(1.0)	0.113
Age 12	857	.08	(1.1)	798	.10	(1.0)	0.822

Weight							
Age 1	2614	−.44	(1.0)	2617	−.45	(1.0)	0.649
Age 2	2464	−.28	(1.0)	2451	−.22	(1.0)	0.133
Age 3	2261	−.26	(1.0)	2188	−.21	(1.0)	0.160
Age 7	1595	−.36	(1.1)	1526	−.29	(1.1)	0.151
Age 10	848	−.24	(1.0)	765	−.13	(1.0)	0.086
Age 12	843	−.21	(1.0)	794	−.11	(1.0)	0.086

Note: *P value based on tests comparing IVF and matched NC groups.

**Table 5:** Number, mean and standard deviation (SD) for internalizing problems (int), externalizing problems (ext) and attention problems (AP) at age 3, 7, 10, and 12 years by rater (mother, father, teacher) for IVF/ICSI twins and matched NC twins.

	IVF/ICSI twins			Matched NC twins			P*
	*N*	Mean	(SD)	*N*	Mean	(SD)	
Age 3							
int mother	2808	4.73	(4.0)	2777	4.48	(3.9)	0.040
int father	2029	4.40	(3.8)	1999	4.28	(3.7)	0.408
ext mother	2799	15.86	(9.6)	2776	15.40	(9.7)	0.147
ext father	2015	14.52	(9.0)	1992	14.50	(9.3)	0.893

Age 7							
int mother	1850	4.91	(4.8)	1810	4.53	(4.8)	0.048
int father	1325	3.72	(4.0)	1274	3.47	(4.1)	0.211
int teacher	1130	5.20	(5.6)	1110	4.69	(5.2)	0.039
ext mother	1879	7.11	(6.3)	1830	7.20	(6.8)	0.721
ext father	1350	6.20	(5.8)	1288	6.32	(6.2)	0.686
ext teacher	1151	4.49	(6.8)	1130	4.51	(7.0)	0.969
AP mother	1888	2.95	(2.9)	1851	2.79	(2.9)	0.126
AP father	1354	2.49	(2.6)	1291	2.42	(2.6)	0.548
AP teacher	1152	5.90	(6.7)	1131	5.51	(6.2)	0.209

Age 10							
int mother	993	5.16	(5.3)	899	4.96	(5.5)	0.514
int father	697	3.51	(4.1)	617	3.72	(4.8)	0.487
int teacher	877	5.57	(5.9)	821	5.05	(5.5)	0.101
ext mother	999	6.25	(6.0)	909	6.52	(6.6)	0.427
ext father	701	4.92	(5.0)	624	5.33	(6.1)	0.271
ext teacher	898	4.85	(7.8)	834	5.13	(7.5)	0.461
AP mother	1005	2.98	(2.9)	913	2.92	(3.1)	0.678
AP father	704	2.41	(2.6)	626	2.53	(2.8)	0.490
AP teacher	900	5.81	(6.5)	836	6.07	(6.8)	0.387

Age 12							
int mother	974	4.60	(5.6)	966	4.52	(5.6)	0.759
int father	690	3.57	(4.5)	658	3.59	(4.6)	0.936
int teacher	392	4.80	(5.5)	355	4.67	(5.4)	0.807
ext mother	979	5.35	(6.0)	966	5.45	(6.3)	0.792
ext father	692	4.40	(5.3)	661	4.59	(5.4)	0.617
ext teacher	400	3.91	(6.5)	360	4.61	(8.6)	0.254
AP mother	988	2.60	(2.9)	972	2.73	(3.1)	0.395
AP father	692	2.28	(2.6)	669	2.34	(2.8)	0.749
AP teacher	402	5.37	(6.2)	360	5.44	(6.5)	0.923

Note: *P value based on tests comparing IVF and matched NC groups.
